# The Chicken HS4 Insulator Element Does Not Protect the *H19* ICR from Differential DNA Methylation in Yeast Artificial Chromosome Transgenic Mouse

**DOI:** 10.1371/journal.pone.0073925

**Published:** 2013-09-04

**Authors:** Eiichi Okamura, Hitomi Matsuzaki, Akiyoshi Fukamizu, Keiji Tanimoto

**Affiliations:** 1 Graduate School of Life and Environmental Sciences, University of Tsukuba, Tsukuba, Ibaraki, Japan; 2 Faculty of Life and Environmental Sciences, University of Tsukuba, Tsukuba, Ibaraki, Japan; 3 Life Science Center of Tsukuba Advanced Research Alliance (TARA), University of Tsukuba, Tsukuba, Ibaraki, Japan; CNRS, France

## Abstract

Mono-allelic expression at the mouse *IGF2/H19* locus is controlled by differential allelic DNA methylation of the imprinting control region (ICR). Because a randomly integrated *H19* ICR fragment, when incorporated into the genome of transgenic mice (TgM), was allele-specifically methylated in somatic, but not in germ cells, it was suggested that allele-discriminating epigenetic signature, set within or somewhere outside of the Tg *H19* ICR fragment in germ cells, was later translated into a differential DNA methylation pattern. To test if the chicken β-globin HS4 (cHS4) chromatin insulator might interfere with methylation imprinting establishment at the *H19* ICR, we inserted the *H19* ICR fragment, flanked by a set of floxed cHS4 core sequences, into a human β-globin locus YAC and generated TgM (*insulated ICR*' TgM). As controls, the cHS4 sequences were removed from one side (*5'HS4-deleted ICR'*) or both sides (*pseudo-WT ICR'*) of the insulated ICR' by *in vivo* cre-loxP recombination. The data show that while maternally inherited transgenic *H19* ICR was not methylated in *insulated ICR*' TgM, it was significantly methylated upon paternal transmission, though the level was lower than in the *pseudo-WT ICR*' control. Because this reduced level of methylation was also observed in the *5'HS4-deleted ICR*' TgM, we speculate that the phenotype is due to VEZF1-dependent demethylation activity, rather than the insulator function, borne in cHS4. Collectively, although we cannot rule out the possibility that cHS4 is incapable of blocking an allele-discriminating signal from outside of the transgene, the epigenetic signature appears to be marked intrinsically within the *H19* ICR.

## Introduction

In genomic imprinting, a subset of mammalian genes are expressed exclusively from the paternally or maternally inherited chromosomes. This imprinted expression is controlled by allele-specific methylation of DNA blocks frequently found in the imprinted loci (DMR; differentially methylated region) [1–4]. The DMRs established during gametogenesis or during the post-fertilization period are called primary and secondary DMRs, respectively. Some primary DMRs acquire DNA methylation imprints in the male germ cells (paternal DMR), while many others become methylated specifically in the female germ cells (maternal DMR). Because *de novo* DNA methyltransferases, DNMT3A and 3L are involved in methylation acquisition in both germ lines [5,6], unique *cis* DNA sequences within or around the DMRs, in addition to hypothetical accessory molecules that confer allele discriminating activity to the DNMTs, are postulated to establish methylation imprinting in the germ line of each gender. To date, however, no apparent difference in *cis* DNA sequences between the paternal and maternal DMRs has been elucidated.

**Figure 1:**
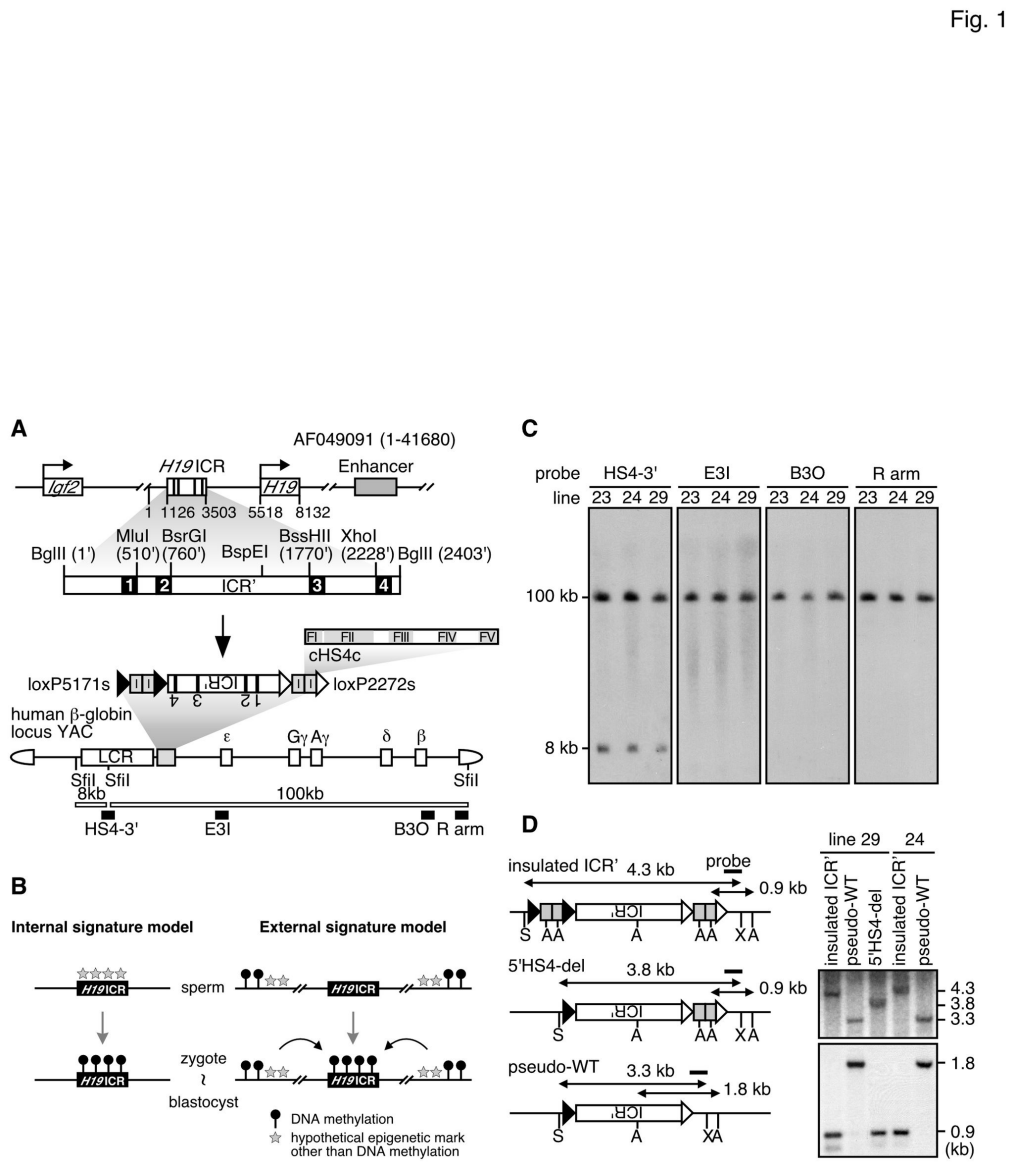
Experimental design. (A) Generation of *Insulated ICR*' transgenic mice (top). Genomic structure of the mouse *Igf2/H19* gene locus. The *H19* ICR fragment used in this study corresponds to nucleotides from 1126 to 3503 in the AF049091 sequences (GenBank). In the enlarged map, restriction enzyme sites (*Mlu*I, *Bsr*GI, *Bss*HII, *Xho*I), which were artificially introduced to facilitate cloning procedure, are shown adjacent to the CTCF binding sites (solid rectangles) of the *H19* ICR (termed the ICR'). Nucleotides are numbered relative to the 5' end (set at 1') of the sequence (bottom). Chicken HS4 core fragment (cHS4c, 237 bp) carries five footprints, FI–FV. Tandem cHS4c fragments (I for insulator, gray rectangles) were floxed by a set of either loxP5171 (solid triangles) or loxP2272 (open) sequences and attached to either ends of the ICR' fragment (termed the insulated ICR'), which was then introduced in reversed orientation 3' to the LCR in the human β-globin locus YAC (A201F4.3 [27]). The entire β-globin locus is contained within two *Sfi*I fragments (8 and 100 kbp). Transgene-specific probes used for long-range structural analysis in (C) are shown as solid rectangles. (B) Two possible models for marking epigenetic signature. The mark can be set inside (left) or outside (right) of the *H19* ICR. (C) Long-range structural analysis of transgenes. DNA from thymus cells was digested with *Sfi*I in agarose plugs, separated by pulsed-field gel electrophoresis, and blots were hybridized separately to probes in (A). (D) (left) Structure of the transgene after *in vivo* cre-*loxP* recombination. Recombination removed the 5' or both (cHS4c)_2_ fragments from the parental insulated ICR' locus and generated either the 5'HS4-del or the pseudo-WT loci, respectively. S, *Sac*I; A, *Ava*II; X, XbaI (right). Tail DNA of each TgM subline was digested with *Xba*I*/Sac*I (top) or *Ava*II (bottom), separated on agarose gels, and blots were hybridized with the probe.

At the imprinted *Igf2/H19* locus, differential DNA methylation of the *H19* DMR is critical for imprinted expression of these genes and therefore, the segment is also called the *H19* ICR (imprinting control region) [1,2] (Figure 1A). The mouse *H19* ICR sequence is CpG-rich and carries four binding sites for CTCF, an insulator protein [7,8]. Preferential DNA methylation of the *H19* ICR is acquired in prospermatogonia [9,10] and is maintained in somatic cells after fertilization. CTCF binding to the *H19* ICR confers enhancer blocking activity to the unmethylated maternal allele, which in turn prevents interaction between the *Igf2* gene and its enhancers, located upstream of the *H19* ICR and downstream of the *H19* gene, respectively. On the methylated paternal allele, a lack of CTCF binding to the *H19* ICR allows the enhancers to alternatively activate the *Igf2* gene. Thus, methylation-sensitive CTCF binding to the *H19* ICR is the molecular basis for imprinted expression at the *Igf2/H19* locus. Furthermore, deletion of the CTCF sites from the maternal *H19* ICR causes its hypermethylation during the post-implantation period, revealing that CTCF-binding also plays an indispensable role in maintenance of the unmethylated state of the maternal *H19* ICR [11]. Importantly, however, the CTCF sites are not required to protect the maternal *H19* ICR from methylation in eggs or pre-implantation embryos. Therefore, the mechanism(s) by which the *H19* ICR attracts and/or is protected from DNA methylation in an allele-specific manner is not yet determined.

As a first step in attempting to identify the sequences responsible for DMR formation in the *H19* ICR, we inserted a 2.9-kb ICR fragment into a human β-globin yeast artificial chromosome (YAC) and generated transgenic mice (TgM) [12]. In somatic cells of these mice, the ICR fragment was hyper- and hypo-methylated when paternally and maternally inherited, respectively, indicating that the 2.9-kb sequence harbored sufficient information to discriminate its parental origin. Surprisingly, however, the transgenic *H19* ICR did not acquire methylation in the testes, demonstrating that establishment of imprinted methylation in germ cells is not essential and it can be established even after fertilization. We therefore presume that gametic and post-fertilization methylation imprinting events are separable, at least in TgM. Because the *H19* ICR in a 147-kb bacterial artificial chromosome (BAC) transgene acquired methylation during spermatogenesis [13], sequences outside the 2.9-kb region were apparently essential for the gametic DNA methylation. For the transgene to be preferentially methylated after fertilization, the 2.9-kb *H19* ICR itself or nearby sequences to the transgene integration sites must be prospectively marked by an epigenetic signature during gametogenesis (Figure 1B). In case the 2.9-kb ICR is intrinsically marked, its signature is unlikely to be DNA methylation [12–15]. On the other hand, if it is externally marked, signature could be a histone modification and/or differential DNA methylation. In either case, the mark must be eventually translated into a differential DNA methylation of the *H19* ICR after fertilization. Histone modifications are candidate epigenetic signatures that could potentially be utilized to mark the parental alleles. In fact, paternally- and maternally-inherited *H19* ICR sequences are associated with different histone marks in somatic cells [16]; of course, those differences may be merely reflecting the allelic DNA methylation status of the *H19* ICR.

The Dnmt3L protein, together with the Dnmt3A plays an essential role in methylation acquisition at the *H19* ICR in prospermatogonia [6]. Interestingly, the Dnmt3L can bind to histone H3 *in vitro* and their association on chromatin is prevented by its dimethyl- or trimethyl-lysine-4 (H3K4me2 or H3K4me3) modifications [17]. In addition, interaction between the Dnmt3A and the histone H3 tail is also sensitive to methylation of the H3K4 [18–20]. Accordingly, it was reported that paternally methylated ICRs, including the *H19* ICR, were associated with unmethylated H3K4 histone marks on the sperm chromatin [21]. Therefore, differential states of histone methylation established during gametogenesis may lead to preferential recruitment of DNA methyltransferase complexes to parental alleles and their differential DNA methylation status, even after fertilization.

In transgenic experiments, insulator sequences are used to 'protect' genes from the spread of heterochromatin. The core region of a prototypical vertebrate insulator sequence, the chicken β-globin hypersensitive site 4 (cHS4) consists of five footprint regions (FI–V). The enhancer blocking activity of the insulator is conferred by CTCF binding to FII [22], while chromatin boundary activity depends on USF1 binding to FIV [23], as well as VEZF1 to FI, FIII, and FV sequences [24]. It has been reported that USF1 can recruit histone methyltransferases, PRMT1 and SET7/9, as well as the histone acetyltransferases, p300/CBP and PCAF [25]. Accordingly, active histone marks, such as acetylated H3/4 and methylated H3K4 are enriched around the FIV site and thereby block invasion of the silencing histone mark, methylated H3K9. In addition, VEZF carries DNA demethylation or protection against methylation activity, which may be involved in maintaining the cHS4 in hypomethylation status [24].

As a first step to identify a primary deposition site(s) of an allele-discriminating signature, i.e. inside or outside the *H19* ICR, we surrounded the ICR fragment with tandemly arrayed cHS4 core sequences (insulated ICR'), which was then used for generating YAC-TgM. Maternally inherited transgenic fragments were not methylated, indicating that placing the cHS4 core sequences on both sides of the *H19* ICR had no apparent effect on its hypomethylation status. Although a paternally inherited and insulated ICR' became significantly methylated after fertilization, the level was seemingly lower than in the control YAC-TgM. Therefore, to examine whether the incomplete paternal methylation is due to blocking of a hypothetical epigenetic signal from outside the *H19* ICR or to demethylation activity of the cHS4 sequences, cHS4 was removed from one or both sides of the *H19* ICR by *in vivo* cre-loxP-mediated recombination (5'HS4-del ICR' and pseudo-WT ICR'). The level of DNA methylation in paternally inherited 5'HS4-del ICR' fragment in somatic cells was similar to that in the insulated ICR' and lower than that in the pseudo-WT ICR' fragments. Therefore, whereas the cHS4 fragment may reduce the DNA methylation level of nearby CpG sites as a consequence of its DNA demethylation activity, it does not interfere with the post-fertilization methylation imprinting establishment at the transgenic *H19* ICR, suggesting that the allele-discriminating epigenetic signature is likely to be marked intrinsically within the *H19* ICR.

## Materials and Methods

### Preparation of ICR' fragment

To facilitate plasmid construction, following two oligonucleotides were phosphorylated, annealed, and ligated to *Sac*I/*Kpn*I-digested pBluescriptII/KS(+) vector, in which two *Bss*HII sites were prospectively disrupted; 5'-CGGGATCCTAGGATCCCGAGCT-3' and 3'-CATGGCCCTAGGATCCTAGGGC-5'. The resultant vector, pBSIIKS(+) _KBABS carried *Kpn*I-*Bam*HI-*Avr*II-*Bam*HI-*Sac*I multi-cloning sites.

Five DNA fragments (I1 trough I5) were PCR-generated by using following primer sets and murine *H19* ICR DNA fragment as a template. Artificially introduced restriction enzyme sites (except for endogenous *Bgl*II sites) and CTCF recognition motifs with their flanking four nucleotides on both sides are underlined and italicized, respectively, and indicated in parentheses.

I1: 5'-GTTC C T A G G C G C C A G A T C TGGCTAGCTTGAGG-3' (*Avr* I I-*Kas* I-*Bgl* I I) and 5'-GGAC T C G A GATGCCGCGTGGTGGCAGTACAATACTACATATTGCTCGG-3' (*Xho* I-*m4*). I2: 5'-TAACC T C G A GTCTGTCCATTTAGCTATA-3' (*Xho*I) and 5'-GAACG C G C G CTACCGCGCGGTGGCAGCATACTCCTATATATCGTGGCC-3' (*Bss* H I I-*m3*). I3: 5'-CGCG C G C G CATCCGTTCCCTTGTTGCA-3' (*Bss*HII) and 5'-GGCT G T A C AGTCTCGTACATCGCAGTC-3' (*Bsr*GI). I4: 5'-CTGCG C G C G C T G T A C ATTCACTGCCGCCGTGCGGCAACCCTGGTCTTTACACACAAAGGA-3' (*Bss*HII-*Bsr*GI-*m2*) and 5'-GGCA C G C G TATCGATTGCGCCAAACCT-3' (*Mlu*I). I5: 5'-GTTTC T C G A G A C G C G TTTTGCTGCCACCACGCGGCAACTCCCGCGTATAAACCCCA-3' (*Xho*I-*Mlu*I-*m1*) and 5'-TGTC C T A G G C G T A C G A G A T C TAGCTCTATCCCAT-3' (*Avr* I I-*Bsi* W I-*Bgl* I I).

The *Avr*II/*Xho*I-digested I1 and *Xho*I/*Avr*II-digested I5 fragments were simultaneously ligated with the *Avr*II-digested pBSIIKS(+) _KBABS vector to generate pKBABS/I1+I5. Then, *Xho*I/*Bss*HII-digested I2 and *Bss*HII/*Mlu*I-digested I4 fragments were simultaneously ligated with the *Xho*I/*Mlu*I-digested pKBABS/I1+I5 vector to make pKBABS/I1+I2+I4+I5. Finally, *Bss*HII/*Bsr*GI-digested I3 fragment was ligated with the *Bss*HII/*Bsr*GI-digested pKBABS/I1+I2+I4+I5 vector to make pICR'. In each cloning step, correctness of DNA sequences were confirmed by DNA sequencing (DDBJ accession no. AB775805).

### Preparation of Targeting Construct and Homologous Recombination in Yeast

A backbone plasmid for targeting construct, pHS1 [26], carried a human β-globin HS1 DNA fragment (from 13299 to 14250 nt; HUMHBB, GenBank) in the pRS306. The following double-stranded DNA (only the upper strand sequences are shown) was subcloned into *Hin*dIII site (at 13769 in HUMHBB) of the pHS1 to generate pHS1/loxP5171-2272: 5'-A A G C T TATAACTTCGTATAGTACACATTATACGAAGTTATG G A T C C T A G G A T C CATAACTTCGTATAGGATACTTTATACGAAGTTATA A G C T T-3' (*Hin* d I I I-*loxP5171*-*Bam* H I-*Avr* I I-*Bam* H I-*loxP2272*-*Hin* d I I I; restriction enzyme sites are underlined and loxP sequences italicized).

Single copy of cHS4 core sequence (238 bp) was PCR-amplified as *Bgl*II-*Bam*HI fragment by using the following primer set and pJC13-1 (generous gift from professor Gary Felsenfeld) as a template: cHS4core-5S2, 5'-AGCCCCA G A T C TCACGGGGACAGCCCCC-3' (*Bgl*II) and cHS4core-3A, 5'-ACTAGTG G A T C CTTTTTCCCCGTATC-3' (*Bam*HI). Following *Bgl*II/*Bam*HI digestion, two copies of the core fragment were subcloned into modified pBluescriptII vector in tandem arrays (i.e. *Bgl*II-*Bam*HI/*Bgl*II-*Bam*HI configuration; pBluescriptG/cHS4 double).

Next, two kinds of tandem cHS4 core sequences were PCR-generated by using above plasmid as a template and two combinations of primer sequences; cHS4core-5S2 and cHS4core-3A-BspEI, 5'-ACTAGTG G A T C C G G ATTCCCCGTATC-3' (*Bam*HI/*Bsp*EI) or cHS4core-5S2-BspEI, 5'-AGCCCCA G A T C T C C G G AGGACAGCCCCC-3' (*Bgl*II/*Bsp*EI) and cHS4core-3A. These two fragments were digested with *Bgl*II/*Bsp*EI or *Bsp*EI/*Bam*HI, respectively, and simultaneously subcloned into *Bam*HI-digested pHS1/loxP5171-2272 to generate pHS1/loxP5171-2272/4xcHS4.

Then, the following double-stranded DNA (only the upper strand sequences are shown) was subcloned into *Bsp*EI site of the pHS1/loxP5171-2272/4xcHS4 to generate pHS1/4xloxP/4xcHS4: 5'- T C C G G A A G C T TATAACTTCGTATAGTACACATTATACGAAGTTATG G A T C C T A G G A T C CATAACTTCGTATAGGATACTTTATACGAAGTTATA A G C T T C C G G A -3' (*Bsp* E I-*Hin* d I I I-*loxP5171*-*Bam* H I-*Avr* I I-*Bam* H I-*loxP2272*-*Hin* d I I I-*Bsp* E I; restriction enzyme sites are underlined and loxP sequences italicized).

Finally, *Avr*II DNA fragment excised from the pICR' was introduced into the *Avr*II site (between the loxP5171 and 2272 sequences) of the pHS1/4xloxP/4xcHS4 to generate pcHS4/ICR' (-) targeting vector.

The targeting DNA was linearized with *Spe*I (at nt position 13670 in HUMHBB) and used for mutagenizing the human β-globin YAC (A201F4.3). Successful homologous recombination in yeast was confirmed by Southern blot analyses with several combinations of restriction enzymes and probes.

### Generation of YAC-TgM

To generate TgM, purified YAC DNA was microinjected into fertilized mouse eggs from CD1 mice (ICR, Charles River Laboratories). Tail DNA from founder offspring was screened first by PCR and then by Southern blotting. The structural analysis of the YAC transgene was performed as described elsewhere [27]. Animal experiments were performed in a humane manner and approved by the Institutional Animal Experiment Committee of the University of Tsukuba. Experiments were conducted in accordance with the Regulation of Animal Experiments of the University of Tsukuba and the Fundamental Guidelines for Proper Conduct of Animal Experiments and Related Activities in Academic Research Institutions under the jurisdiction of the Ministry of Education, Culture, Sports, Science and Technology of Japan.

### Methylation analysis by Southern blotting

Genomic DNA was prepared from tail-tip cells (1- to 2-weeks-old) or whole testes (2-month-old) of TgM using standard procedures. DNA was firstly digested by *Bam*HI (for 3' portion of the *H19* ICR'), *Bam*HI+*Bsr*GI (middle portion), or *Bam*HI+*Sac*I (cHS4 portion), then by methylation-sensitive *Hha*I enzymes. DNA was separated on an agarose gel, and transferred to a nylon membrane. The membrane was hybridized with the α-^32^P-labeled probes and subjected to X-ray film autoradiography.

### Bisulfite sequencing

Genomic DNA was extracted from tail-tip cells of TgM (1- to 2-weeks-old, pool of 1-2) and digested with *Xba*I. The DNA was treated with sodium bisulfite using the EZ DNA Methylation Kit following the manufacturer’s instructions (Zymo Research). Transgenic ICR-specific nested PCR, PCR product cloning, and sequence analysis were performed as described previously [12,15]. PCR primers used are:

ICR-MA-5S1: 5'-GAATTTGAGGATTATGTTTAGTGG-3',
ICR-MA-3A17: 5'-AACTTTAAAAAAAAACTATCCTCC-3',
ICR-MA-5S15: 5'-TTGTATGGTTATAGTATTGTTATG-3',
BGLB-MA-3A5: 5'-AACTTAACTCATTCCCTACACAAC-3',
ICR-MA-5S2: 5'-TTAAGGATTAGTATGAATTTTTGG-3',
ICR-MA-3A2: 5'-AACAATACTAAATCTACCTAAAAC-3',
ICR-MA-5S14: 5'-TTGAATGGATAGTGAGTAGGAAAG-3',
BGLB-MA-3A3: 5'-TTTATCCAAATACTAAATAACACC-3'.

## Results

### Generation of YAC-TgM

Imprinting control region of the *Igf2/H19* locus in mouse is located from -4.4 to -2.0 kb of transcription start site of the *H19* gene (*H19* ICR; Figure 1A, top). To facilitate plasmid DNA construction, artificial restriction enzyme sites were introduced into the 2.4-kb *H19* ICR fragment near the four CTCF binding sites. This modified sequence is referred to as the "ICR'", hereafter (GenBank accession no. AB775805). In order to shelter this fragment from the spread of possible epigenetic signaling, such as DNA methylation and/or histone modifications (Figure 1B, right), tandemly-arrayed chicken HS4 core sequences, (cHS4c)_2_ were ligated to either side of the ICR' fragment. The cHS4c sequence used here carries all the footprint sites (FI–V) required for full insulator activity [28,29]. In order to delete the (cHS4c)_2_ sequences afterward as a rigorous control that would be transgene integration site independent, 5' and 3' (cHS4c)_2_ fragments were surrounded by a pair of *loxP5171* or *loxP2272* sequences, respectively (Figure 1A, bottom). This "insulated ICR'" fragment was inserted at 3' to LCR in the human β-globin locus YAC, which was then used to generate TgM. Long-range structural analyses of the transgene by Southern blotting revealed that three YAC-TgM lines (lines 23, 24 and 29) carried intact, single copies of the transgene (Figure 1A, C, and not shown).

To initiate *in vivo* cre-*loxP* recombination, parental YAC-TgM (insulated ICR', lines 24 and 29) were crossed with cre-TgM, and offspring were analyzed for recombination by Southern blotting (Cre-F0, data not shown). Pups that carried the recombined transgene were then crossed with non-Tg animals to remove the cre-containing transgene. Recombination removed the 5' (termed "5'HS4-del" allele) or both 5' and 3' ("pseudo-WT" allele) (cHS4c)_2_ fragments from the parental allele, which was confirmed by Southern blot analysis (Figure 1D and data not shown).

### DNA Methylation Status of the H19 ICR and the (cHS4c)_2_ Sequences in Insulated ICR' TgM

**Figure 2:**
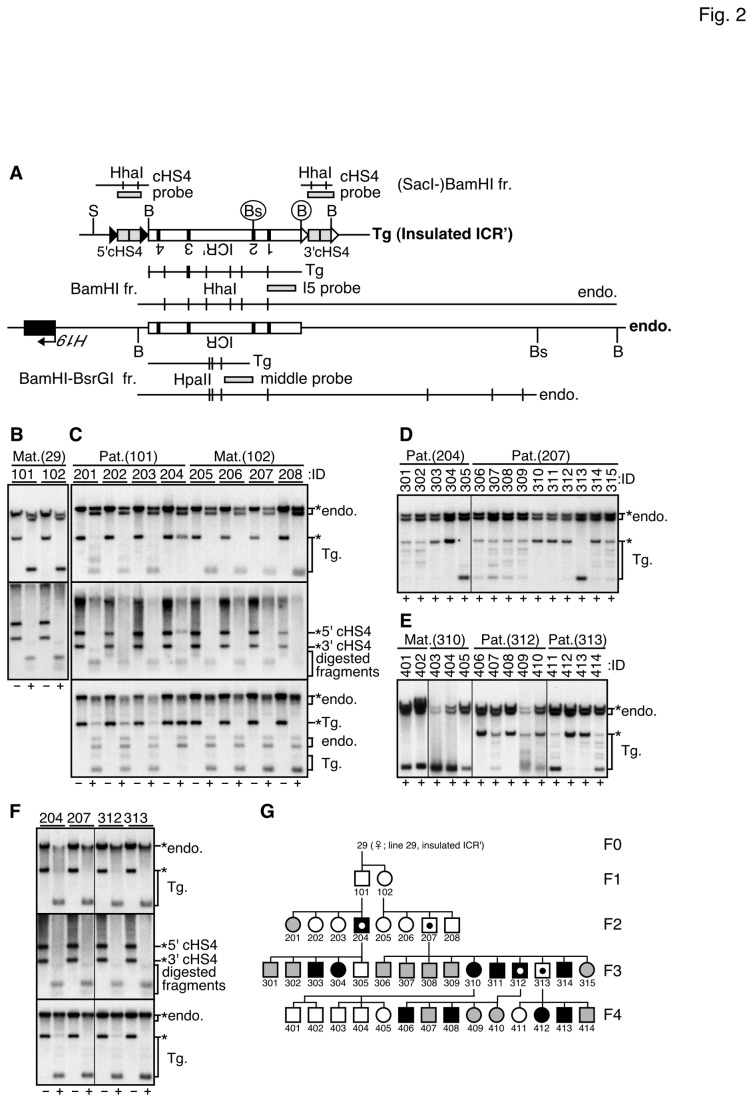
DNA methylation status of the insulated ICR' fragment in somatic and germ cells (line 29). (A) Partial restriction enzyme map of the Insulated ICR' transgene. Fragments from the transgenic (Tg) and endogenous (endo.) *H19* ICR can be distinguished by their sizes because of a transgene-specific *Bam*HI (B) or *Bsr*GI (Bs) sites (circled). Methylation-sensitive *Hha*I or *Hpa*II sites in the *Sac*I (S)-*Bam*HI, *Bam*HI, or *Bam*HI-*Bsr*GI fragments (horizontal lines) are displayed as vertical lines. Probes are shown as gray rectangles. (B, C) Genomic DNA from a tail-tip of TgM (*Insulated ICR*', F1 (B) and F2 (C) generations), inheriting the transgene either paternally (Pat.) or maternally (Mat.) was analyzed by Southern blotting (upper panel). DNA was digested with *Bam*HI in the presence (+) or absence (-) of *Hha*I and analyzed by the I5 probe shown in (A) to analyze 3' portion of the transgenic *H19* ICR'. Parental fragments are marked by asterisks. (middle) DNA was digested with *Bam*HI+*Sac*I in the presence (+) or absence (-) of *Hha*I and analyzed by the cHS4 probe. (bottom) DNA was digested with *Bam*HI+*Bsr*GI in the presence (+) or absence (-) of *Hpa*II and analyzed by the middle probe to analyze middle portion of the *H19* ICR. (D, E) Methylation status of the 3' portion of the *H19* ICR in F3 (D) and F4 (E) generations was analyzed (by I5 probe). (F) Genomic DNA from testis of the TgM was analyzed by I5 (top panel), cHS4 (middle), or middle (bottom) probes. (G) Pedigree depicting a paternally-methylated insulated ICR' transgene. Male and female (hemi-zygous) transgenic individuals are denoted as squares and circles, respectively. In the Southern blot data of 3' portion of the transgenic *H19* ICR' sequences, uncut (top), completely-cut (bottom), and other (intermediate) bands were quantified by Phosphorimager and methylation status of each sample was determined as follows. When ratio of uncut/total (top+intermediate+bottom) bands exceeds 50%, the sample was defined as hyper-methylated and represented by solid marks. When ratio of completely-cut/total bands exceeds 50%, the sample was defined as hypo-methylated (open marks). In other cases, the sample was defined as partially-methylated (shaded marks). Testis samples in (F) were obtained from male individuals marked by dots.

We first examined the DNA methylation status of the *H19* ICR fragment in tail-tip somatic cells of the insulated ICR' TgM (Figure 2). Because the *Bam*HI and *Bsr*GI sites are unique to the transgene, the transgenic and endogenous ICRs can be distinguished by Southern blotting (Figure 2A). We analyzed cytosine methylation in the recognition sequences of the methylation-sensitive *Hha*I enzyme using the "I5 probe", which detects the 3' portion of the transgenic *H19* ICR (corresponding to 5' portion of the endogenous *H19* ICR) sequences (Figure 2A). In the F1 and F2 generations (top panels in Figure 2B and C), the maternally-inherited transgenic *H19* ICR' (Tg) was hardly methylated, while it was variably methylated upon paternal inheritance. Because half of the endogenous *H19* ICR fragment (presumably the paternal allele) was resistant to *Hha*I digestion (endo, Figure 2B–E), the paternally inherited endogenous allele was likely to be highly methylated in the same samples. This allele-preferential methylation pattern was also observed in the following generations (Figure 2D and E). To assess methylation status in the middle portion of the *H19* ICR, the DNA was digested with the methylation-sensitive *Hpa*II enzyme and analyzed using the "middle probe" (bottom panel in Figure 2C) for Southern blotting. The DNA methylation status in this region of the transgenic *H19* ICR' fragment was similar to that seen in the 3' portion of the fragment.

We next examined the methylation status of the (cHS4c)_2_ fragments by Southern blot analysis. The (cHS4c)_2_ sequences attached to either 5'- or 3'-ends of the ICR' can be distinguished by their sizes when genomic DNA is digested with *Bam*HI and *Sac*I (Figure 2A). Southern blot analysis using a combination of *Hha*I and the cHS4 probe revealed that these fragments were hypo-methylated irrespective of whether they are paternally or maternally inherited (middle panel in Figure 2C).

To determine the methylation status of the transgene in more detail, we conducted bisulfite sequencing of the DNA regions covering the CTCF sites 1/2 (3' portion) of the *H19* ICR' and the (cHS4c)_2_ sequences (Figure S1). Based on the Southern blot results (Figure 2C and D), highly (No. 311 and 312), partially (No. 307 and 308), and poorly methylated (No. 313) DNA samples from the paternally-inherited TgM, as well as that from the maternally-inherited TgM (No. 205 and 206) were individually pooled and subjected to analysis. Consistent with the Southern blot results, *H19* ICR' sequences were variably methylated in paternally-inherited transgenes, while they were hypomethylated upon maternal transmission. In contrast, the (cHS4c)_2_ sequences were infrequently methylated regardless of their parental inheritance, suggesting that the (cHS4c)_2_ sequences harbor anti-methylation protective or demethylation activities.

Finally, we examined the methylation status of the transgene in the testis DNA of the insulated ICR' TgM by Southern blotting (Figure 2F). The transgenic *H19* ICR' (both 3' and middle regions), as well as the (cHS4c)_2_ sequences were unmethylated. The weak methylated Tg band seen in the No. 204 and 312 individuals (those inheriting the transgene paternally) probably represent somatic cell DNA contamination, because the band was visible neither in the No. 207 nor 313 individuals (inheriting the transgene maternally). These results demonstrated that methylation imprinting in the transgene was established after fertilization, as has been shown in our previous reports [12,15,30].

By integrating the methylation status of individuals determined by Southern blot analyses of the *H19* ICR' transgene (determined by I5 probe), pedigree of the insulated ICR' TgM (line 29) was depicted in Figure 2G. The results demonstrated that the transgene was hypomethylated when maternally inherited. In contrast, it was methylated after paternal transmission, although the level of which was variable, indicating that the paternal phenotype was not fully penetrant.

We also analyzed two other insulated ICR' lines (lines 23 and 24 in Figures S2 and S3, respectively) and obtained essentially the same results.

### DNA methylation status of the H19 ICR sequence in pseudo-WT ICR' TgM

**Figure 3:**
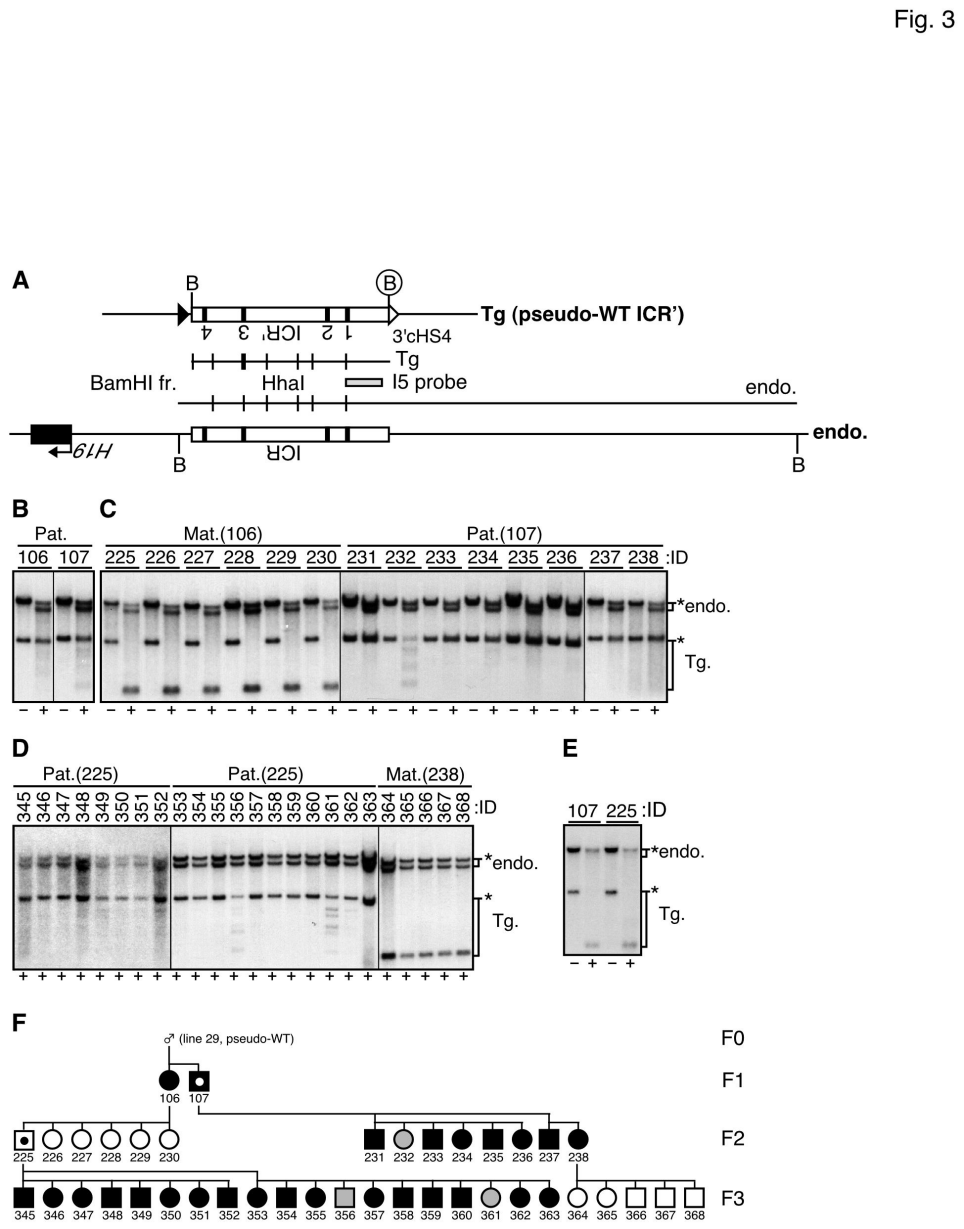
Methylation status of the pseudo-WT ICR' fragment in somatic and germ cells (line 29). (A) Partial restriction enzyme map of the ICR' (pseudo-WT) transgene. (B–D) Southern blot analysis of the transgene in F1-3 generations. Genomic DNA was prepared from a tail-tip of TgM (*pseudo-WT* TgM) and the 3' portion of the transgenic *H19* ICR' was analyzed (*Bam*HI+*Hha*I) as described in the legend to Figure 2B and C. (E) Genomic DNA was prepare from testis of the TgM and the 3' portion of the transgenic *H19* ICR' was analyzed (*Bam*HI+*Hha*I) as described in the legend to Figure 2B and C. (F) Pedigree depicting a paternally-methylated pseudo-WT ICR' transgene is shown as described in the legend to Figure 2G.

In our previous reports [12], we did not observe such a variegated DNA methylation level in the paternally inherited transgenic *H19* ICR fragment. We therefore decided to ask if the phenotype observed in the insulated ICR' transgene was elicited by the flanking (cHS4c)_2_ sequences or by inserting the artificial enzyme sites in to the *H19* ICR' sequences. To that end, we removed both 5' and 3' (cHS4c)_2_ sequences from the insulated ICR' transgene by *in vivo* cre-*loxP* recombination to generate pseudo-WT ICR' allele (Figure 1D) and examined their DNA methylation statuses at the same chromosomal integration site (Figure 3 and Figure S4). In somatic cells of the pseudo-WT TgM, methytlation level of 3' portion of the paternally inherited *H19* ICR' in somatic cells (Figure 3B–D and Figure S4B and D) was apparently more uniform than that of the insulated ICR' TgM (Figure 2 and Figures S2 and S3) and most of them exhibited hypermethylation. DNA methylation level of the maternally inherited pseudo-WT transgene (Figure 3C, D and Figure S4A–C), as well as that in the testis (Figure 3E and Figure S4E) were low, which was indistinguishable from those in the insulated ICR' TgM (Figure 2 and Figures S2 and S3).

These results demonstrated that the (cHS4c)_2_ fragments bordering the *H19* ICR' sequence on both sides somehow interfered with methylation acquisition or carry a demethylation activity at least at the paternally inherited *H19* ICR' sequence, which caused its variegated DNA methylation.

### DNA methylation status of the H19 ICR sequence in 5'HS4-del ICR' TgM

**Figure 4:**
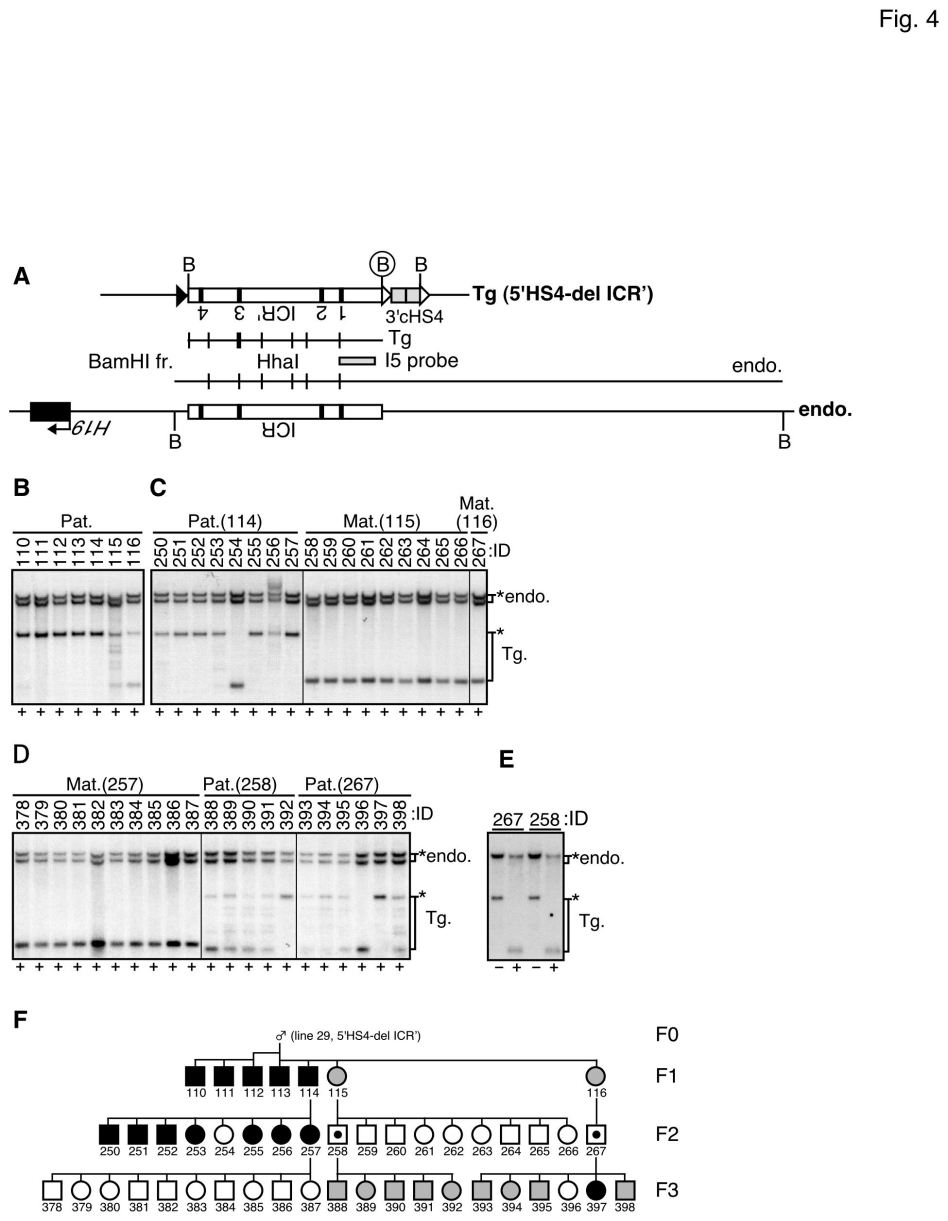
Methylation status of the 5'HS4-del ICR' fragment in somatic and germ cells (line 29). (A) Partial restriction enzyme map of the ICR' (5'HS4-del) transgene. (B–D) Southern blot analysis of the transgene in F1-3 generations. Genomic DNA was prepared from a tail-tip of TgM (*5'HS4-del* TgM) and the 3' portion of the transgenic *H19* ICR' was analyzed (*Bam*HI+*Hha*I) as described in the legend to Figure 2B and C. (E) Genomic DNA was prepared from testis of the TgM and the 3' portion of the transgenic *H19* ICR' was analyzed (*Bam*HI+*Hha*I) as described in the legend to Figure 2B and C. (F) Pedigree depicting a paternally-methylated 5'HS4-del ICR' transgene is shown as described in the legend to Figure 2G.

We hypothesized that an allele-discriminating epigenetic signature might be found outside the *H19* ICR' and yet still affects *H19* ICR' function (Figure 1B, right). Chromatin boundary activity of the chicken HS4 sequences has previously been suggested to be elicited by impeding the spread of repressive epigenetic signatures, either suppressive (H3K9me) histone modifications and/or DNA methylation of chromatin [23]. By employing such activities, (cHS4c)_2_ fragments lying on both sides of the *H19* ICR' could interfere with methylation imprinting establishment by preventing encroachment of any epigenetic signature (parental mark) on both sides of the *H19* ICR', resulting in variable methylation levels of the paternally inherited *H19* ICR', as was seen in the insulated ICR' TgM. If this were the case, full methylation imprinting might be restored by removing either one of the (cHS4c)_2_ fragment from the insulated *H19* ICR'. To test this concept, we screened for TgM (line 29) that had undergone partial *in vivo* cre-*loxP* recombination, and found one having only the 5'(cHS4c)_2_ sequence deleted (5'HS4-del ICR', Figures 1D and 4A). Southern blot analysis of these mutant animals revealed that paternally inherited 5'HS4-del ICR' transgene in the tail somatic DNA was still variably methylated (Figure 4B–D), while a maternally inherited transgene (Figure 4C and D), as well as DNA from testis (Figure 4E) were hypomethylated. Because this methylation pattern was similar to that observed in the insulated ICR' TgM (Figures 2G, 4F, S2F, S3F, and 5), we conclude that the variegated DNA methylation at the paternal *H19* ICR' was likely to be caused by demethylation activity, but not the insulator activity, of the (cHS4c)_2_ sequence. 

**Figure 5:**
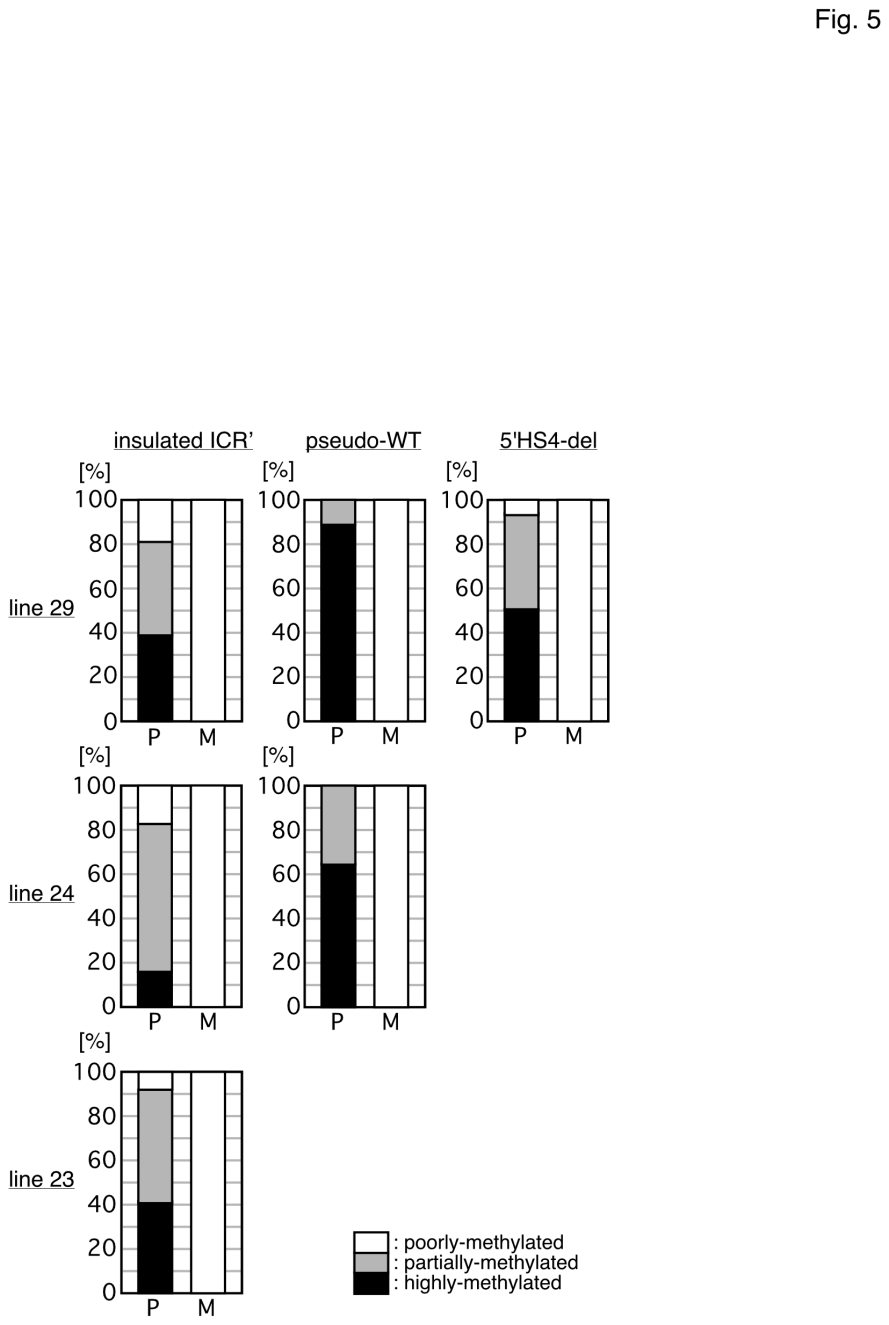
Summary of the methylation levels in each transgenic mouse line. The vertical axis of the bar graph represents the percentage of individuals showing distinct degree of DNA methylation at the transgenic H19 ICR' fragment (3' portion), which was summarized from pedigrees of each TgM line. For each 100% stacked column, the number of individuals analyzed in each pedigree (Fig. 2-4 and Fig. S2-S4) was set at 100 (%) and DNA methylation levels were divided into three categories (highly-, partially- and poorly-methylated). P, paternal transmission; M, maternal transmission.

### Discussion

Differential DNA methylation of the *H19* ICR, which is normally established during gametogenesis, is essential to normal embryonic development in the mouse [31,32] and human [33], in part because it is utilized to discriminate parental alleles and to control mono-allelic expression of the *Igf2* and *H19* genes. Curiously, however, we and others have reported that gametic methylation of the *H19* ICR is not mandatory to establish its DMR status and that this status can surprisingly be established even after fertilization [12–14], even in the situation where both paternal and maternal alleles coexist in a single cell, perhaps by propagating some unknown epigenetic signature that is set in the germ cells. Although the 2.9-kb *H19* ICR fragment in TgM was unable to instruct its own methylation acquisition in sperm (our unpublished result), the ~140-kb BAC was capable of doing so [13]. Deductively then, establishment of germ-line methylation appears to require sequences lying outside the boundaries of the 2.9-kb *H19* ICR fragment. It is therefore reasonable that the transgenic *H19* ICR also requires a hypothetical epigenetic mark that resides outside the 2.9-kb *H19* ICR region on the paternal chromosome to control post-fertilization methylation imprinting (Figure 1B, right). Because these randomly integrated *H19* ICR fragments (into the mouse genome) acquired methylation imprinting at chromosomal sites where a DMR was not found nearby [15], we concluded that the primary epigenetic mark in this case may not be DNA methylation.

While most genome-associated histones are replaced by protamines during spermatogenesis, some defined regions, including the *H19* ICR, remain associated with histones [21,34,35]. We therefore hypothesized that histone modifications within, or outside of (not necessarily restricted to β-globin sequences), the *H19* ICR during spermatogenesis might direct allele-specific DNA methylation after fertilization.

Di- and tri-methylated lysine 9 methylation of histone H3 (H3K9me2 and -me3, respectively) are involved in heterochromatin protein 1 (HP1) recruitment and heterochromatin propagation. Because the H3K9me3 modification facilitates DNA methylation in non-mammalian model organisms [36] and the same mark is associated with methylated (paternal) *H19* ICR regions [16,37,38], we presumed that the H3K9me3 epigenetic mark might be set during spermatogenesis and then translated into subsequent DNA methylation after fertilization in the TgM. Because it was reported that H3K9me3 modification was completely depleted in the *H19* ICR during primordial germ cells (PGC) development [10] and was not found at the *H19* ICR in spermatocytes or round/elongating spermatids [16], we surmised that this repressive histone mark may be set outside the *H19* ICR and only later spread into the ICR sequences in later stages of spermatogenesis, prior to fertilization. In addition, because the *H19* ICR is marked by dimethyl H3K9 in sperm, Nakamura et al. proposed that the paternal *H19* ICR is protected from genome-wide DNA demethylation during early embryogenesis through its’ interaction with the PGC7 protein mediated by the H3K9me2 tail [35]. In accord with this notion, the level of DNA methylation at the *H19* ICR in the paternal allele was diminished in somatic cells of *Pgc7*-null mutant mice. Therefore, the H3K9me2 modification found in mature sperm might also serve as an allele-specific DNA methylation signal at the *H19* ICR after fertilization.

It has been shown previously that constitutive hyper-acetylation of H3K9 at the cHS4 insulator excludes its further modification by di- and tri-methylation [23]. Furthermore, cHS4 is purported to inhibit encroachment of H3K9 trimethylation by recruiting the H3K4me3 activating mark into nearby chromatin in a USF-dependent manner [25]. To ask whether two copies of the cHS4 fragment (1.2 kb x 2), (ChβGI)_2_, carrying a similar number of CpGs as the *H19* ICR as well as two CTCF-binding sites would generate proper DMR activity, Szabo et al. replaced the endogenous *H19* ICR with the (ChβGI)_2_ in mice [39]. The (ChβGI)_2_ sequence did not acquire methylation neither in sperm nor in eggs, indicating that the fragment lacks *cis* sequences that can direct DNA methylation acquisition during gametogenesis. In addition, the mutant sequence was hypomethylated in somatic cells regardless of its’ paternal origin, indicating that the (ChβGI)_2_ did not acquire an epigenetic mark that can be subsequently translated into differential DNA methylation status. We therefore decided to test whether post-fertilization methylation imprinting at the *H19* ICR is disturbed by placing the cHS4 chromatin boundary at both ends by prohibiting the hypothetical encroachment of repressive histone modifications from the *H19* ICR neighborhood.

When inherited maternally, the transgenic *H19* ICR as well as the cHS4 sequences were hypomethylated in somatic cells of the insulated ICR' TgM (Fig. 2 and Fig. S1-S3). When paternally inherited, the cHS4 portion of the insulated ICR' transgene was again unmethylated, as was expected from the result of Szabo et al. [39]. However, the *H19* ICR segment of the transgene was significantly methylated, although its level was somehow lower than that of a pseudo-WT transgene (Fig. 5). We therefore envisioned two possible explanations for the observed phenotypes: 1) the epigenetic signature (such as the H3K9me3) was set outside the transgenic fragment and its spread into the *H19* ICR sequence was obstructed by the cHS4 chromatin insulator, and thus its transformation to complete DNA methylation was partially prohibited; 2) although the epigenetic signature was set within the *H19* ICR, its conversion into the appropriate DNA methylation status or perhaps DNA methylation *per se* was inhibited by the surrounding cHS4 sequences. To distinguish between these two possibilities, we removed the cHS4 sequences only from the 5' side of the transgene (5'HS4-del ICR'), because, in this situation, the hypothetical external epigenetic signature would presumably spread into the *H19* ICR. In those TgM, we found a similar (somewhat reduced) level of *H19* ICR methylation upon paternal transmission, a result most consistent with the second possibility. However, it is also possible that the signature spreads only uni-directionally, *eg.* the H3K9me mark may spread in from 3' side of the ICR', where histone association in the sperm has been reported in the endogenous human β-globin locus [40]. It is also formally possible that allele-restricted transcription may occur across the *H19* ICR [10,41] at the ectopic sites and this "external signal" might guide post-fertilization methylation imprinting, since the cHS4 insulator is not capable of blocking such transcriptional run through. In addition, although mRNAs for Usf1, Usf2, and Vezf1 are shown to be present in the embryonic testes at E15.5 (GenitoUrinary Development Molecular Anatomy Project; http://www.gudmap.org.), we cannot exclude the possibility that cHS4 insulator activity may not be fully established at the time when this hypothetical epigenetic signal spreads (i.e. the latest stage of spermatogenesis), leading to variegated DNA methylation at the insulated *H19* ICR.

Following the report by Szabo et al. [39], a similar experiment was repeated using the mutant cHS4 sequence, (mChβGI)_2_, lacking the USF and VEZF1 binding sites, both required for chromatin boundary activity of the insulator [42]. In those mutant mice, the sequences were partially methylated (32%) in sperm germ cells, suggesting that, although the CpGs in cHS4 can potentially acquire DNA methylation, the USF and/or VEZF1 sites protect much of the cHS4 from DNA methylation in the sperm. Because it has been reported that VEZF1 sites are capable of mediating protection from *de novo* DNA methylation [24], we postulated that placing VEZF sites (in cHS4) on one or both sides of the *H19* ICR fragment in the YAC-TgM caused its partial demethylation in the paternal allele. Alternatively, the H3K4me3 mark associated with the USF site in cHS4 might cause the hypomethylation of nearby sequences, since Dnmt3L preferentially interacts with unmethylated H3K4 and induces *de novo* DNA methylation [17].

We assume that establishment of methylation imprinting at the *H19* ICR during gametogenesis and early embryogenesis are separable events [15]; the latter is normally masked because of the former at the endogenous locus. Based on the results reported here, we infer that the epigenetic signature to direct post-fertilization methylation imprinting is likely to be set within the *H19* ICR and that it does not require "spread-in" of additional information from the neighborhood. However, because methylation acquisition at the endogenous *H19* ICR during spermatogenesis requires sequences outside of the 2.9-kb region [12,13,15], the spread of external epigenetic marks into the *H19* ICR may be the basis for its gametic DNA methylation. Unfortunately, we were unable to examine the effect of inserting cHS4 on gametic DNA methylation of the *H19* ICR, because transgenic *H19* ICR does not acquire DNA methylation in germ cells [12]. If gametic methylation is affected by introducing the cHS4 on both sides of the endogenous *H19* ICR, that would provide significant insight into the underlying molecular mechanisms of methylation imprinting establishment at the endogenous *H19* ICR. In addition, by obstructing methylation acquisition at the endogenous *H19* ICR during gametogenesis, we may be able to assess a functional role, if any, for the post-fertilization methylation activity at the endogenous locus.

### Supporting Information

Figure S1 **Methylation analysis of the insulated ICR' transgene (line 29) by bisulfite sequencing.** (A) Schematic representation of transgene-specific DNA amplification. Methylation status of two distinct regions of the transgene (3' portion of the *H19* ICR' and the cHS4) was determined. The position of the primers used for nested PCR is shown by arrowheads: a, ICR-MA-5S1; b, ICR-MA-3A17; c, ICR-MA-5S15; d, BGLB-MA-3A5; e, ICR-MA-5S2; f, ICR-MA-3A2; g, ICR-MA-5S14; h, BGLB-MA-3A3. Solid and open arrowheads are for the first and second round PCR reactions, respectively. (B) (top) Enlarged map of a part of the Figure S1A (lower). Genomic DNA was prepared from tail tip of individuals, each inheriting the transgenes either maternally (ID 205 and 206) or paternally, in which methylation levels of the 3' portion of the *H19* ICR' were determined to be high (311 and 312), partial (307 and 308) and poor (313) by Southern blotting in the Figure 2. DNA was digested with *Xba*I, treated with sodium bisulfite, and amplified by nested PCR. PCR products were subcloned and DNA sequences of the region corresponding to the enlarged map were determined. Each horizontal row represents a single DNA template molecule. Methylated (solid circles) and unmethylated (open circles) CpG motifs are shown. Vertical arrows indicate the locations of the *Hha*I enzyme recognition sites.
(TIF)

Figure S2 **Methylation status of the insulated ICR' fragment in somatic and germ cells (line 23).** (A–D) Southern blot analysis of the transgene in F1-4 generations. Genomic DNA was prepared from a tail-tip of TgM (*Insulated ICR*', line 23) and its methylation status (the 3' portion of the *H19* ICR (upper) and the cHS4 (lower)) was analyzed as described in the legend to Figure 2B and C. (E) Genomic DNA was prepared from testis of the TgM and the 3' (left) and middle (right) portions of the *H19* ICR or the cHS4 were analyzed by Southern blotting as described in the legend to Figure 2B and C. (F) Pedigree depicting a paternally-methylated insulated-ICR' transgene is shown as described in the legend to Figure 2G.
(TIF)

Figure S3 **Methylation status of the insulated ICR' fragment in somatic and germ cells (line 24).** (A–D) Southern blot analysis of the transgene in F1-4 generations. Genomic DNA was prepared from a tail-tip of TgM (*Insulated ICR*', line 24) and its methylation status (the 3' (top) and middle (bottom) portions of the *H19* ICR or the cHS4 (middle)) was analyzed as described in the legend to Figure 2B and C. (E) Genomic DNA was prepared from testis of the TgM and the 3' (top) and middle (bottom) portions of the *H19* ICR or the cHS4 were analyzed by Southern blotting as described in the legend to Figure 2B and C. (F) Pedigree depicting a paternally-methylated insulated ICR' transgene is shown as described in the legend to Figure 2G.
(TIF)

Figure S4 **Methylation status of the pseudo-WT ICR' fragment in somatic and germ cells (line 24).** (A–D) Southern blot analysis of the transgene in F1-4 generations. Genomic DNA was prepared from a tail-tip of TgM (*pseudo-WT*, line 24) and the 3' portion of the *H19* ICR was analyzed as described in the legend to Figure 2B and C. (E) Genomic DNA was prepared from testis of the TgM and the 3' portion of the *H19* ICR was analyzed by Southern blotting as described in the legend to Figure 2B and C. (F) Pedigree depicting a paternally-methylated insulated ICR' transgene is shown as described in the legend to Figure 2G.
(TIF)
