# Supplementary figures and images for: The Chicken HS4 Insulator Element Does Not Protect the H19 ICR from Differential DNA Methylation in Yeast Artificial Chromosome Transgenic Mouse
Source: PLoS One. 2013 Sep 4;8(9):e73925. doi: 10.1371/journal.pone.0073925 (PMC3762768; doi:10.1371/journal.pone.0073925)

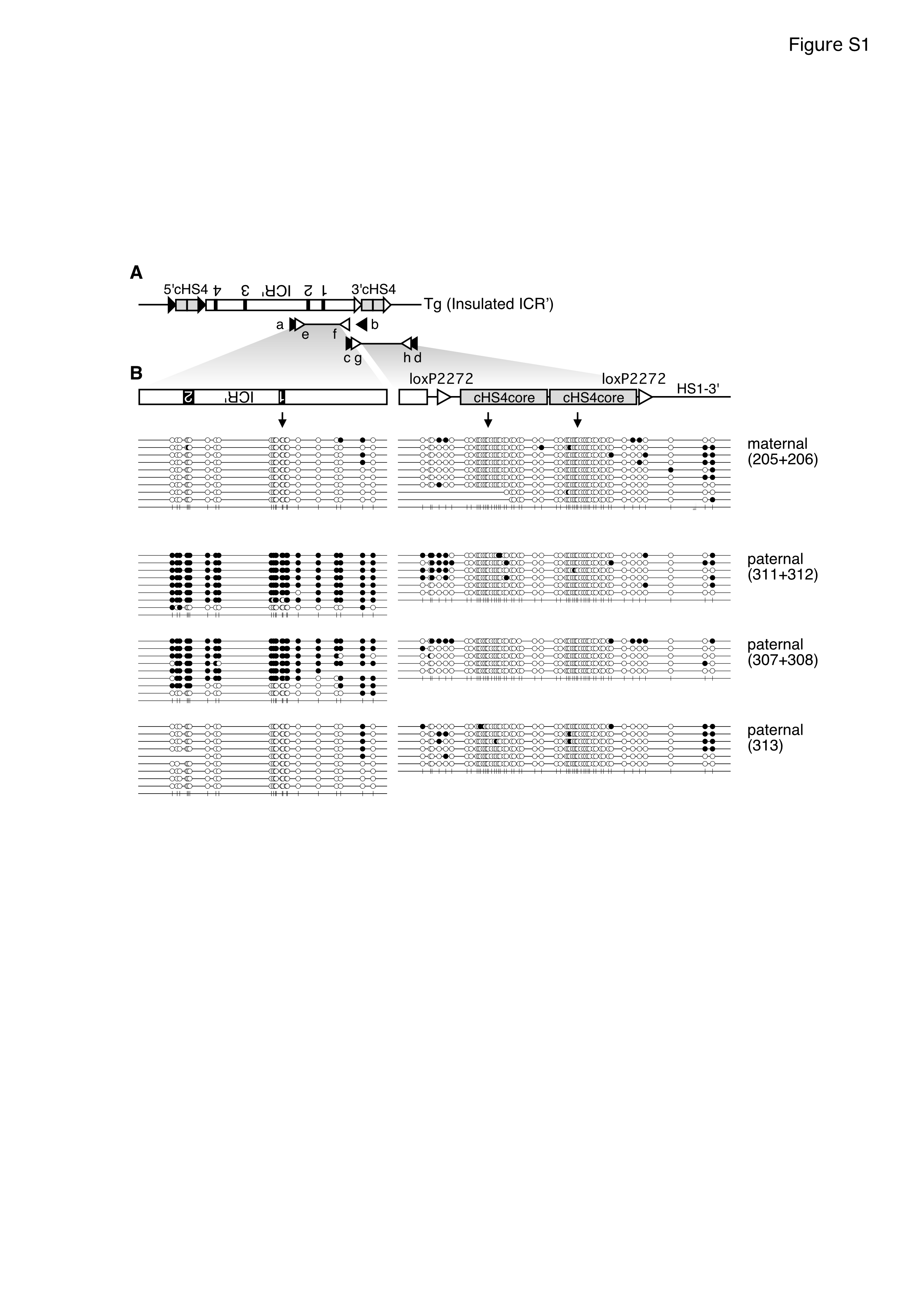

Supplement: Figure S1 — Methylation analysis of the insulated ICR' transgene (line 29) by bisulfite sequencing. (A) Schematic representation of transgene-specific DNA amplification. Methylation status of two distinct regions of the transgene (3' portion of the H19 ICR' and the cHS4) was determined. The position of the primers used for nested PCR is shown by arrowheads: a, ICR-MA-5S1; b, ICR-MA-3A17; c, ICR-MA-5S15; d, BGLB-MA-3A5; e, ICR-MA-5S2; f, ICR-MA-3A2; g, ICR-MA-5S14; h, BGLB-MA-3A3. Solid and open arrowheads are for the first and second round PCR reactions, respectively. (B) (top) Enlarged map of a part of the Figure S1A (lower). Genomic DNA was prepared from tail tip of individuals, each inheriting the transgenes either maternally (ID 205 and 206) or paternally, in which methylation levels of the 3' portion of the H19 ICR' were determined to be high (311 and 312), partial (307 and 308) and poor (313) by Southern blotting in the Figure 2. DNA was digested with XbaI, treated with sodium bisulfite, and amplified by nested PCR. PCR products were subcloned and DNA sequences of the region corresponding to the enlarged map were determined. Each horizontal row represents a single DNA template molecule. Methylated (solid circles) and unmethylated (open circles) CpG motifs are shown. Vertical arrows indicate the locations of the HhaI enzyme recognition sites. (TIF) [file pone.0073925.s001.tif]

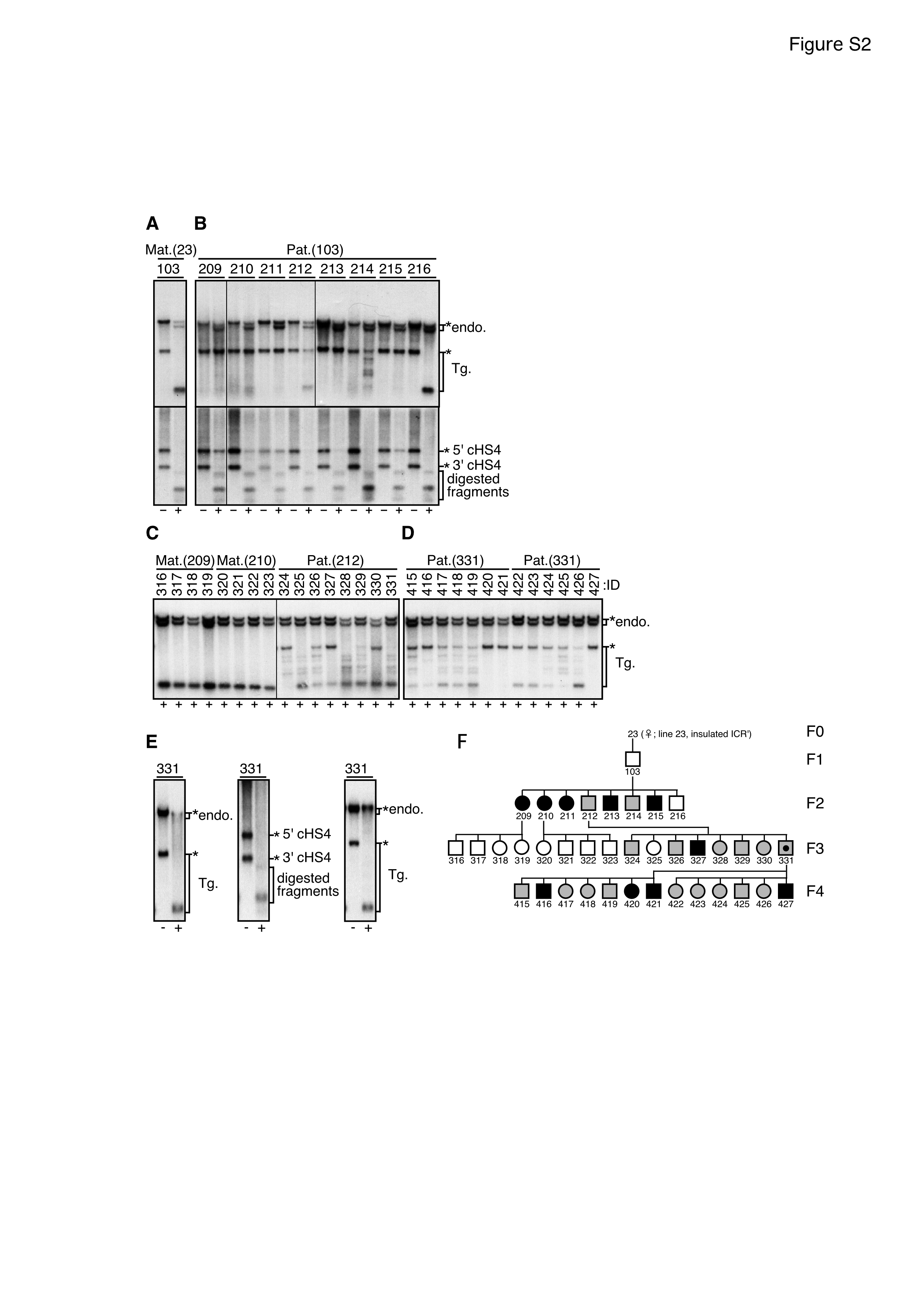

Supplement: Figure S2 — Methylation status of the insulated ICR' fragment in somatic and germ cells (line 23). (A–D) Southern blot analysis of the transgene in F1-4 generations. Genomic DNA was prepared from a tail-tip of TgM (Insulated ICR', line 23) and its methylation status (the 3' portion of the H19 ICR (upper) and the cHS4 (lower)) was analyzed as described in the legend to Figure 2B and C. (E) Genomic DNA was prepared from testis of the TgM and the 3' (left) and middle (right) portions of the H19 ICR or the cHS4 were analyzed by Southern blotting as described in the legend to Figure 2B and C. (F) Pedigree depicting a paternally-methylated insulated-ICR' transgene is shown as described in the legend to Figure 2G. (TIF) [file pone.0073925.s002.tif]

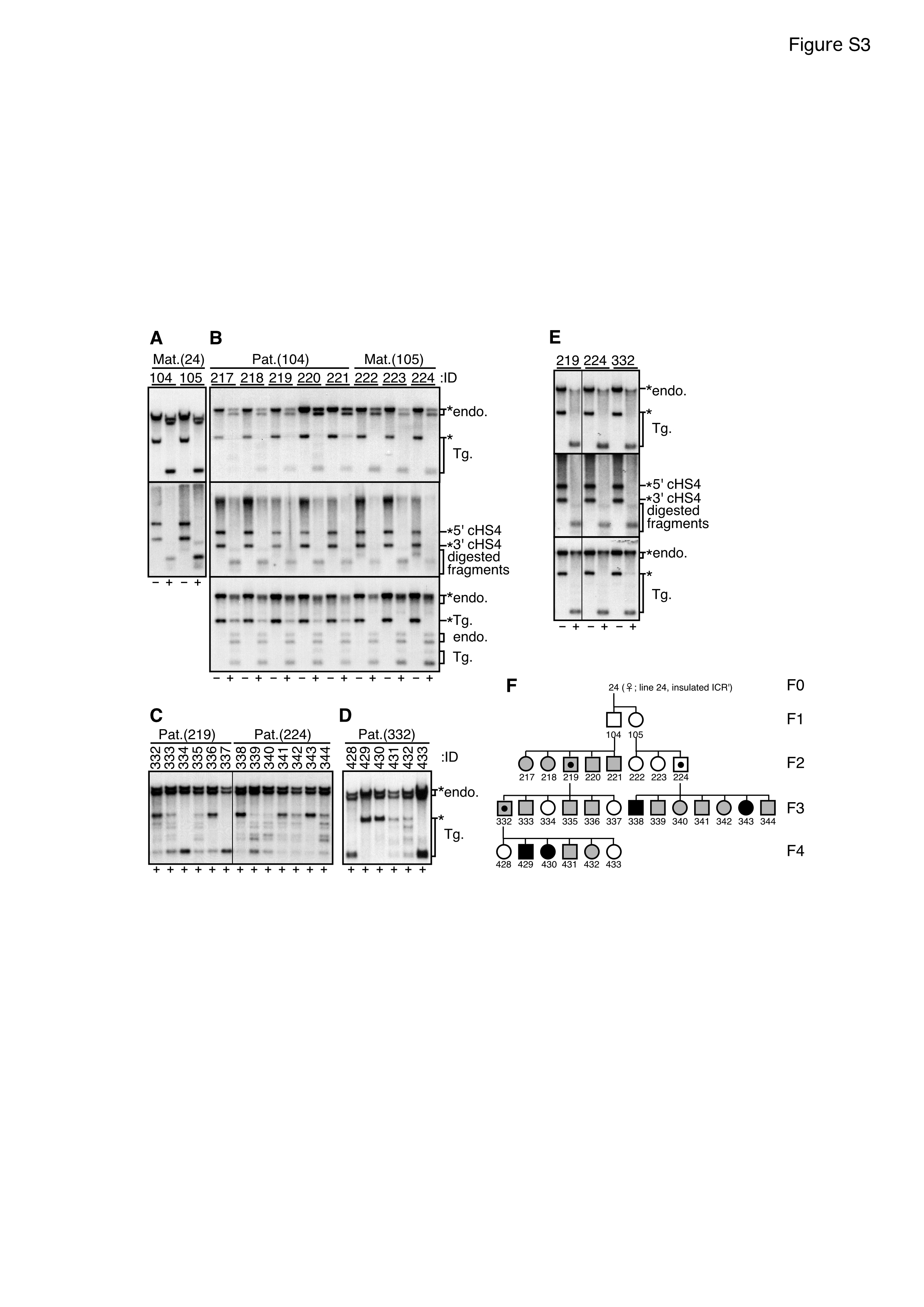

Supplement: Figure S3 — Methylation status of the insulated ICR' fragment in somatic and germ cells (line 24). (A–D) Southern blot analysis of the transgene in F1-4 generations. Genomic DNA was prepared from a tail-tip of TgM (Insulated ICR', line 24) and its methylation status (the 3' (top) and middle (bottom) portions of the H19 ICR or the cHS4 (middle)) was analyzed as described in the legend to Figure 2B and C. (E) Genomic DNA was prepared from testis of the TgM and the 3' (top) and middle (bottom) portions of the H19 ICR or the cHS4 were analyzed by Southern blotting as described in the legend to Figure 2B and C. (F) Pedigree depicting a paternally-methylated insulated ICR' transgene is shown as described in the legend to Figure 2G. (TIF) [file pone.0073925.s003.tif]

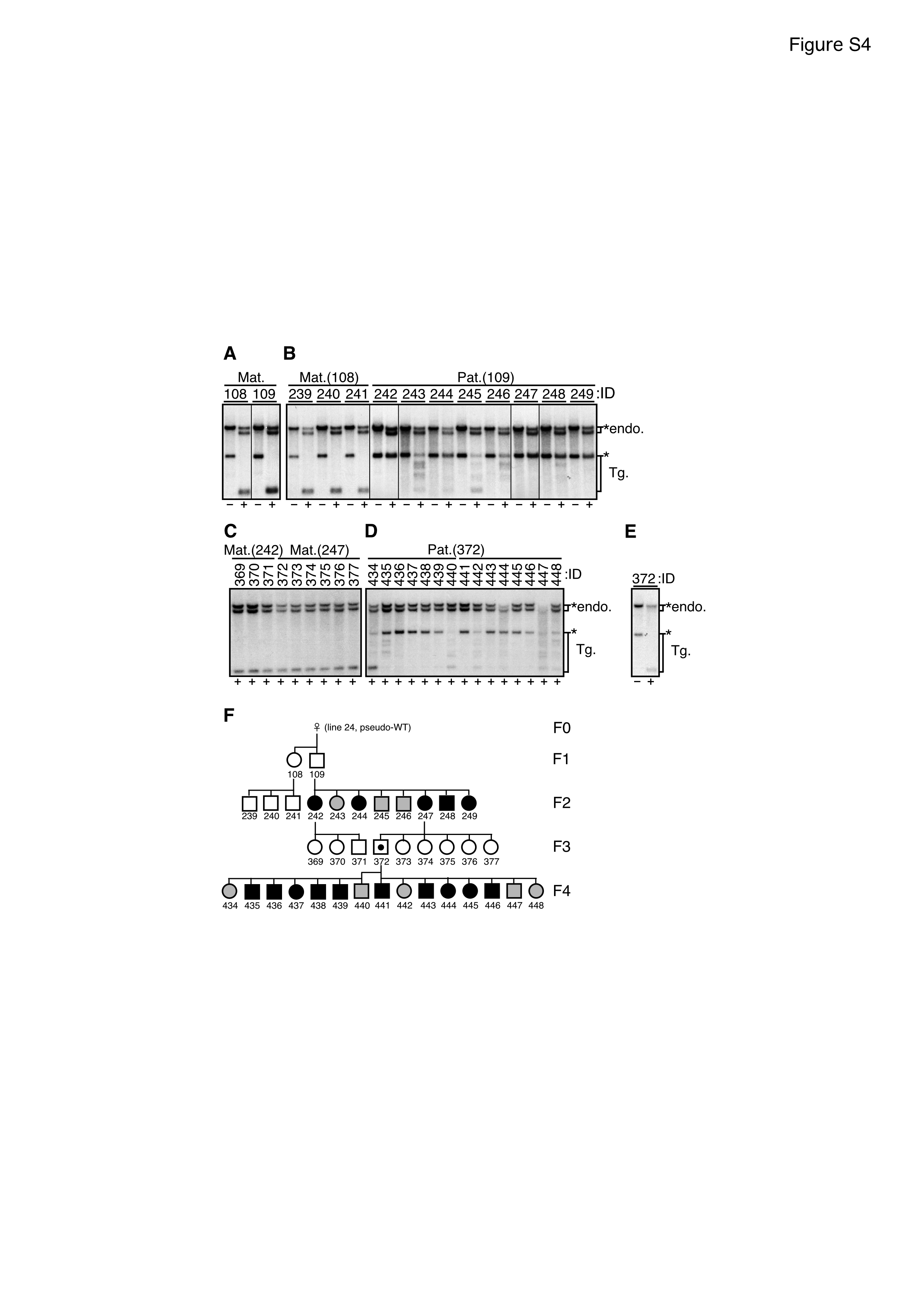

Supplement: Figure S4 — Methylation status of the pseudo-WT ICR' fragment in somatic and germ cells (line 24). (A–D) Southern blot analysis of the transgene in F1-4 generations. Genomic DNA was prepared from a tail-tip of TgM (pseudo-WT, line 24) and the 3' portion of the H19 ICR was analyzed as described in the legend to Figure 2B and C. (E) Genomic DNA was prepared from testis of the TgM and the 3' portion of the H19 ICR was analyzed by Southern blotting as described in the legend to Figure 2B and C. (F) Pedigree depicting a paternally-methylated insulated ICR' transgene is shown as described in the legend to Figure 2G. (TIF) [file pone.0073925.s004.tif]
